# 615. Burn Camp Attendance - A Vital Extension of the Burn Care Continuum

**DOI:** 10.1093/jbcr/irag033.429

**Published:** 2026-04-06

**Authors:** Ruth B Rimmer, Curt C Bay, Daniel W Chacon, Nicole Cuevas, Kevin N Foster

**Affiliations:** Care Plans For Life, Phoenix, AZ; AT Still University, Phoenix, AZ; Phoenix Society for Burn Survivors, Fresno, CA; Arizona Burn Foundation, Phoenix, AZ; Diane & Bruce Halle Arizona Burn Center - Valleywise Health, Phoenix, AZ

## Abstract

**Introduction:**

Research has shown that a disfiguring burn injury in childhood often results in social isolation and bullying. Burn camp has been recommended as a rehabilitative experience for burn-injured youth but few studies have asked participants for their opinion of the actual benefits they receive by attending. This study sought to give voice to survivors on if and how burn camp helps them with their recovery.

**Methods:**

Burn-injured youth were invited to voluntarily complete a survey asking them why they would recommend burn camp to a burn-injured child and why attendance is beneficial. The survey included 10 items outlining the potential value of attending camp.

**Results:**

Participants included burn injured youth (n = 64), mean age of 14.7 years, SD (±1.6), female (n = 29), male (n = 35), Caucasian (17%), Hispanic (55%), and African American (17%), average of 5.5 years at burn camp, with 75% reporting visible burn scars. Top reported benefit of burn camp for both males & females was “making friends/building connections.” For males #2 was no judgement zone & #3 learning new things, with females #2 was helping me accept my scars & #3 tied between increasing my confidence & no judgement zone. Other benefits included trying and learning new things, being a part of a community, supportive counselors. Getting time away from home and being outside had the lowest levels of endorsement.

**Conclusions:**

This study endorses burn camp attendance as a safe space for burn-injured youth that provides a supportive environment for making friends with peers that accept their scars and understand what they have been through. They report that acceptance in a judgement free zone helps to build confidence, accept their scars and increase social support.

**Applicability of Research to Practice:**

Burn care professionals should be encouraging attendance at burn camp as this study suggests that attendance has transformative benefits that go far beyond a burn-injured youth’s physical recovery.

**Funding for the study:**

Arizona Burn Foundation.

